# Identification of subject-specific responses to footwear during running

**DOI:** 10.1038/s41598-023-38090-0

**Published:** 2023-07-12

**Authors:** Fabian Horst, Fabian Hoitz, Djordje Slijepcevic, Nicolas Schons, Hendrik Beckmann, Benno M. Nigg, Wolfgang I. Schöllhorn

**Affiliations:** 1 Department of Training and Movement Science, Institute of Sport Science, Johannes Gutenberg-University Mainz, Mainz, Germany; 2 Biomedical Engineering, Schulich School of Engineering, University of Calgary, Calgary, AB Canada; 3 Human Performance Laboratory, Faculty of Kinesiology, University of Calgary, Calgary, AB Canada; 4 Institute of Creative Media Technologies, Department of Media & Digital Technologies, St. Pölten University of Applied Sciences, St. Pölten, Austria

**Keywords:** Machine learning, Diagnostic markers, Risk factors

## Abstract

Placing a stronger focus on subject-specific responses to footwear may lead to a better functional understanding of footwear’s effect on running and its influence on comfort perception, performance, and pathogenesis of injuries. We investigated subject-specific responses to different footwear conditions within ground reaction force (GRF) data during running using a machine learning-based approach. We conducted our investigation in three steps, guided by the following hypotheses: (I) For each subject x footwear combination, unique GRF patterns can be identified. (II) For each subject, unique GRF characteristics can be identified across footwear conditions. (III) For each footwear condition, unique GRF characteristics can be identified across subjects. Thirty male subjects ran ten times at their preferred (self-selected) speed on a level and approximately 15 m long runway in four footwear conditions (barefoot and three standardised running shoes). We recorded three-dimensional GRFs for one right-foot stance phase per running trial and classified the GRFs using support vector machines. The highest median prediction accuracy of 96.2% was found for the subject x footwear classification (hypothesis I). Across footwear conditions, subjects could be discriminated with a median prediction accuracy of 80.0%. Across subjects, footwear conditions could be discriminated with a median prediction accuracy of 87.8%. Our results suggest that, during running, responses to footwear are unique to each subject and footwear design. As a result, considering subject-specific responses can contribute to a more differentiated functional understanding of footwear effects. Incorporating holistic analyses of biomechanical data is auspicious for the evaluation of (subject-specific) footwear effects, as unique interactions between subjects and footwear manifest in versatile ways. The applied machine learning methods have demonstrated their great potential to fathom subject-specific responses when evaluating and recommending footwear.

## Introduction

Debates in sports biomechanics have discussed the effects of footwear on sports performance, comfort perception, and injury risks^1,2^. From these debates, footwear designs emerged that were aimed to reduce speculated risk factors of running-related injuries (e.g., excessive pronation or high impact forces). The effects of such footwear designs, however, remain elusive as contradictory findings regarding their influence on injury risks and biomechanics are frequently reported^3,4^. Some studies, for instance, showed that a shoe’s midsole hardness affected ground reaction force (GRF) variables (e.g., loading rates, impact forces)^5,6^, while other authors reported no (or even opposing) effects^7–9^.

One possible explanation for these contradictory findings could be related to methodological limitations. Current research strategies commonly focus on average responses to footwear (e.g., using estimates of central tendencies like mean values from groups of individuals). This approach, however, neglects footwear-related effects on individual subjects^2,10^. This is a limitation as differences between anatomy^11,12^, history of previous injuries^13^, and milage^13^ were reported across individuals. Runners, therefore, have varying responses to different footwear designs^14^. A notion that is supported by the concept of movement signatures: the finding of unique movement patterns for each individual in (barefoot) walking^15,16,17^ and running with one’s personal shoe^18,19^. Consequently, group-based approaches may have led to an incomplete functional understanding of how footwear affects a subject’s (unique) movement^14,20,21^.

Research strategies with a stronger focus on subject-specific responses to footwear designs have been discussed^22^. These research strategies are categorised into *single-subject*^22^ and *functional group*^2,23,24^ -based approaches. Either approach needs to consider holistic biomechanical data (i.e., several multi-dimensional and time-continuous variables) to map subject-specific responses to footwear because previous findings have shown that movement signatures^25,26^ and responses to footwear^4^ manifest in multiple interacting variables.

Machine learning models offer a holistic data analysis approach as they can process several multi-dimensional, time-continuous variables (e.g., three-dimensional lower-body joint angles)^10^. Consequently, no pre-selection of single time-discrete variables from time-continuous variables is required (Fig. 2). The potential of machine learning-based approaches has been demonstrated in previous studies investigating the uniqueness of movement patterns for each individual in (barefoot) walking^15,16,17^ and running with one’s personal shoe^18,19,27^. Machine learning models, therefore, are suited to analyse the multi-faceted interactions in the responses of subjects to footwear interventions^28–31^.

This work aimed to explore the uniqueness of individual responses to different footwear conditions using a machine learning-based classification via support vector machines (SVMs). We conducted our investigation in three steps, guided by the following hypotheses:

I. For each subject x footwear combination, unique GRF patterns can be identified (30 subjects × 4 footwear conditions = 120 unique patterns).
II. For each subject, unique GRF characteristics can be identified across various footwear conditions (30 subjects = 30 unique patterns).
III. For each footwear condition, unique GRF characteristics can be identified across various subjects (4 footwear conditions = 4 unique patterns).

## Materials and methods

### Subjects and ethics statement

Thirty healthy, physically active male subjects (Age: 20–28 years; Height: 1.80–1.90 m; Mass: 71.4–100.0 kg) that were free of lower extremity injuries participated in the study. Prior to any testing, subjects provided written informed consent. All experimental procedures were conducted in accordance with the Declaration of Helsinki and were approved by the ethical committee of the medical association Rhineland-Palatinate in Mainz (Germany).

### Experimental protocol

Subjects performed running trials at their preferred (self-selected) speed along a level, 15 m long runway in four shod and one barefoot condition. The four running shoes were the New Balance Minimus, Adidas Adistar Boost, ON Cloudsurfer, and the subject’s personal running shoe. Footwear conditions were counterbalanced across subjects. For each footwear condition, subjects performed running trials until ten records with foot strikes on the force plate were available. Prior to data collection, subjects performed twenty familiarisation runs in each new footwear condition. Data acquisition took place on a single day in an indoor laboratory.

### Data acquisition

Per running trial, three-dimensional GRFs were recorded at a frequency of 1,000 Hz for one right-foot stance phase using a floor-embedded force plate (Kistler, Type 9287CA, Switzerland) located halfway along the runway. The recording was processed within the LabView 2010 (National Instruments, USA) framework. Subjects were instructed to focus on a gender-neutral face emoji (i.e., simple, open eyes and a flat, closed mouth) on the opposing wall of the laboratory to direct their visual attention away from targeting the force plate and ensure a natural run with an upright body position.

### Data processing

The recorded three-dimensional GRFs, mediolateral (GRF_ML_), anteroposterior (GRF_AP_), and vertical (GRF_V_), were filtered using a second order Butterworth bidirectional low-pass filter at a cut-off frequency of 50 Hz. Stance phase was determined based on the filtered vertical GRF using a threshold value of 10 N. Each GRF signal was time-normalised to 101 data points, corresponding to 100% stance phase. GRF signals were normalised to the body weight, measured separately for each footwear condition. Data processing was performed exclusively within MATLAB 2021b (MathWorks, USA).

### Data analysis

For data analysis, the barefoot and the three standardized shoe conditions were utilized. The personal shoe condition was excluded from further analysis due to similarities observed with the standardized shoe conditions in certain subjects. A total of 1200 GRF recordings were classified using SVMs^32^. The L2-regularised L2-loss support vector classification of the Liblinear Toolbox 1.4.1^33^ with a linear kernel function was applied. The regularisation parameter C was experimentally determined using a grid search within the range of C = 2^−5^, 2^−4.75^, …, 2^15^ prior model training / testing. GRF signals were min–max normalised to the range of 0 to 1 and concatenated before being passed to the SVM models. The grid search and determination of minima and maxima were conducted exclusively based on recordings that were included in the training data.

Three classification tasks were tested: (1) *subject x footwear*, where each subject-footwear combination represented one of 120 (30 subjects × 4 footwear conditions) possible classification outcomes (hypothesis I); (2) *subject*, where each subject represented one of 30 possible classification outcomes (hypothesis II); and (3) *footwear*, where each footwear represented one of 4 possible classification outcomes (hypothesis III).

#### Performance evaluation

**Figure 1:**
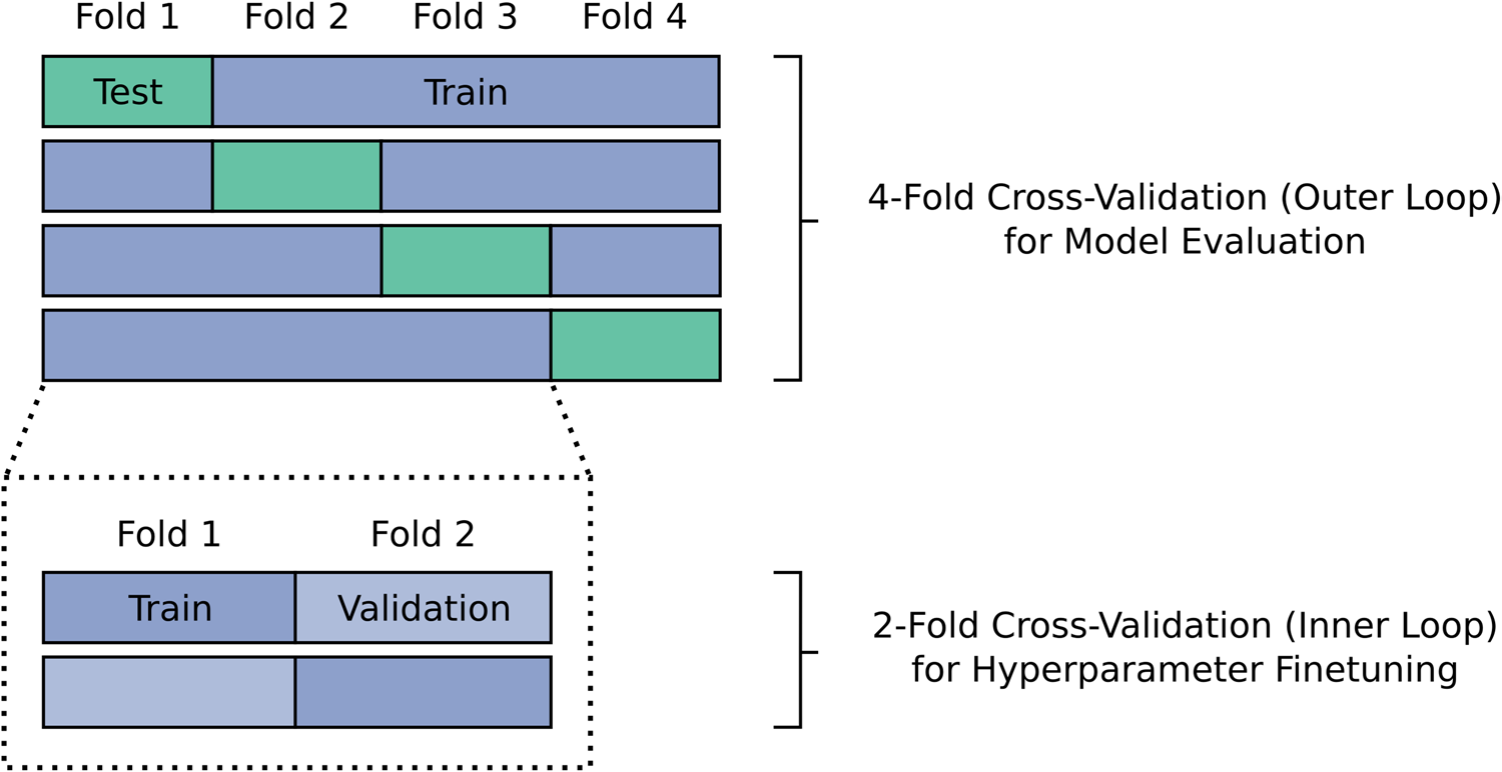
Illustration of the nested k-fold cross-validation scheme used in the experiments. The outer loop, which consists of four folds, evaluates the performance of the model by iteratively evaluating one fold as test data. Within each iteration of the outer loop, an inner two-fold cross-validation is performed on the training data from the other loop to estimate the optimal hyperparameters.

For all classification tasks, a stratified four-fold cross-validation was used to evaluate the classification performance (Fig. 1). Each individual recording was part of the test data once. For the subject x footwear classification, recordings from each combination of subject and footwear condition were distributed equally among the cross-validation folds. For the subject classification and the footwear classification, we ensured for each fold that the recordings of each footwear condition (in the subject classification) and each subject (in the footwear classification) were either part of the training *or* the test data.

The individual classification performances were compared to the zero-rule baseline, which refers to the theoretical accuracy obtained by assigning class labels according to the prior probabilities of the classes. Specifically, the target labels were set to the class with the largest cardinality in the training data, corresponding to 0.8%, 3.3%, and 25.0% for subject x footwear, subject, and footwear, respectively.

#### Relevance score evaluation

Layer-wise relevance propagation (LRP)^34^ was used to decompose the predictions of the trained SVM models into relevance scores for each value *i* of the corresponding input vector. The relevance scores *R*_*i*_ were calculated based on the product of each value *x*_*i*_ of the input vector *x* and the weight *w*_*i*_ of the weight vector *w* of the trained SVM models:

$$R_{i} = x_{i} *w_{i}$$

Relevance scores indicate which information was used by the SVM model for its prediction. Positive scores represent variables supporting the classification, while negative scores represent variables speaking against a given classification. For this work, the ground truth class labels were decomposed, and only positive input relevance scores were analysed^34^. To ensure better comparability between individual explanations, positive relevance scores were normalised to their respective maximum.

All data analysis was performed within the MATLAB 2021b (MathWorks, USA) framework.

#### Statistical evaluation

For the statistical evaluation of differences between the time-continuous GRFs of the four footwear conditions, which also served as input for the SVMs, we conducted an one-way analysis of variance (ANOVA) with statistical parametric mapping (SPM). The evaluation was performed using the SPM1D package for MATLAB provided by Pataky (2012)^35^. We used α = 0.05 as decision-making significance threshold and depicted significant signal regions as grey-shaded areas in Figs. 5 and 6.

**Figure 2:**
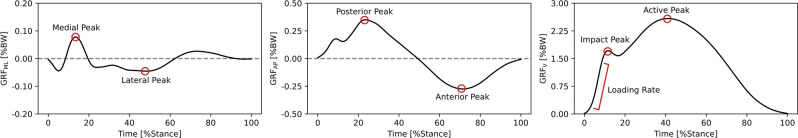
The mediolateral, anteroposterior, and vertical ground reaction force, along with the corresponding time-discrete variables (highlighted in red) used to assess statistical differences between footwear conditions. For the peaks we compared the force and the temporal occurrence during stance. The ground reaction force components were normalized to body weight (%BW) and stance phase (%stance).

As a supplementary part to the statistical evaluation, we followed a prevailing approach employed in biomechanical footwear research: Specifically, we derived a set of time-discrete variables from the time-continuous GRF variables (Fig. 2). The Kruskal–Wallis-test, serving as non-parametric alternative to an ANOVA, was conducted to examine differences between the four footwear conditions. In cases of statistically significant differences, we run the post-hoc analysis with the Dwass-Steel-Critchlow-Flinger-test for pairwise comparisons. The supplementary evaluation of the time-discrete GRF variables was performed in the JAMOVI framework^36^. The results of the supplementary evaluation can be found in Supplementary Table 1.

## Results

### Performance evaluation

**Figure 3:**
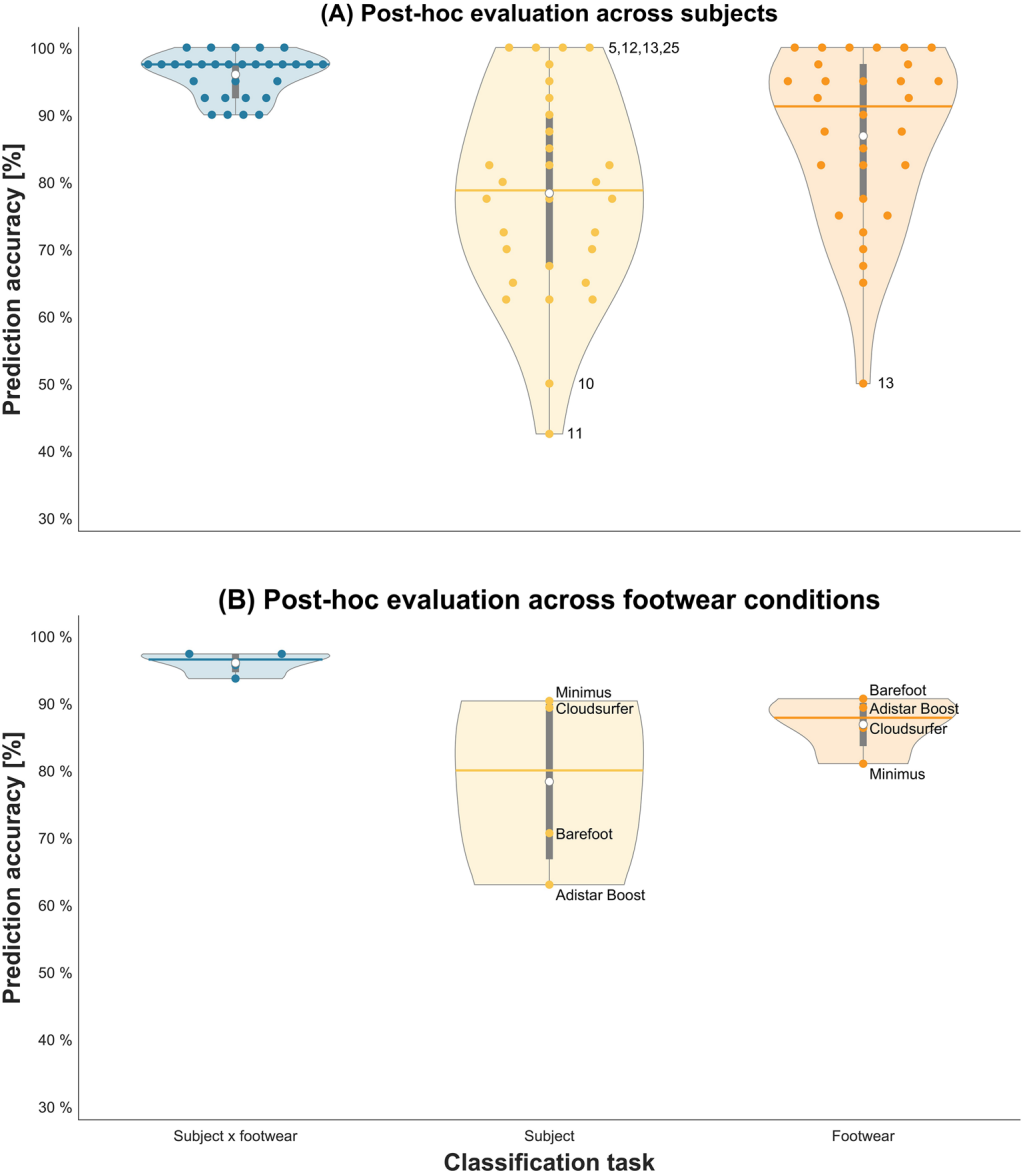
Post-hoc evaluation of the support vector machine model performance as a function of individual (**A**) subjects and (**B**) footwear conditions. Performance evaluation of the models trained for the three employed classification tasks: subject x footwear (in blue on the left), subject (in yellow in the middle), and footwear (in orange on the right). The task-specific zero-rule baseline values are 0.8% (subject x footwear classification), 3.3% (subject classification), and 25.0% (footwear classification). The prediction accuracy is shown as violin plots with median (solid line), mean (white dot), interquartile range from Q1 to Q3 (grey bar), and individual values (coloured dots) of the prediction accuracy for individual (**A**) subjects and (**B**) footwear conditions. The numbers in the violin plots highlight subject IDs that are discussed in sections "Performance evaluation", "Subject classification (across footwear conditions)", and "Footwear classification (across subjects)". The figure was created using the MATLAB code provided by Bechtold et al. (2022)^37^.

For all classification tasks, the median prediction accuracy across the four-fold cross-validation was superior to the task-specific zero-rule baseline. The highest prediction accuracy was found for the subject x footwear classification task (hypothesis I), with a median value of 96.2% (120 times higher than the respective zero-rule baseline of 0.8%). For the subject classification task (hypothesis II), the median accuracy was 80.0% (24 times higher than the zero-rule baseline of 3.3%). For the footwear classification task (hypothesis III), the median accuracy was 87.8% (3.5 time higher than the zero-rule baseline of 25.0%). In addition to the fold-wise performance evaluation (Supplementary Fig. 1), the prediction accuracy of the SVM models was also evaluated in a post-hoc manner according to individual subjects (Fig. 3A) and footwear conditions (Fig. 3B).

Most individuals were correctly identified across different footwear conditions, with a few individuals (e.g., subject 5, 12, 13, 25) that were consistently identified across all footwear conditions. For a few individuals (e.g., subject 10, 11) the SVM was unable to effectively model individual characteristics across all footwear conditions, resulting in a lower classification performance at a range of 40–50%. For the subject classification task, matching movement patterns to individuals (across footwear conditions) was most accurate when individuals ran in the New Balance Minimus (accuracy of 90.3%) and ON Cloudsurfer (accuracy of 89.3%). The worst classification results were obtained with the Adidas Adistar Boost (accuracy of 63.0%).

For the footwear classification task, running barefoot was identified most accurately with 90.7%. The New Balance Minimus was recognised with the lowest accuracy (81.0%). Interestingly, the accuracy at which footwear conditions were correctly identified varied greatly across individuals (50–100%).

### Relevance score evaluation

**Figure 4:**
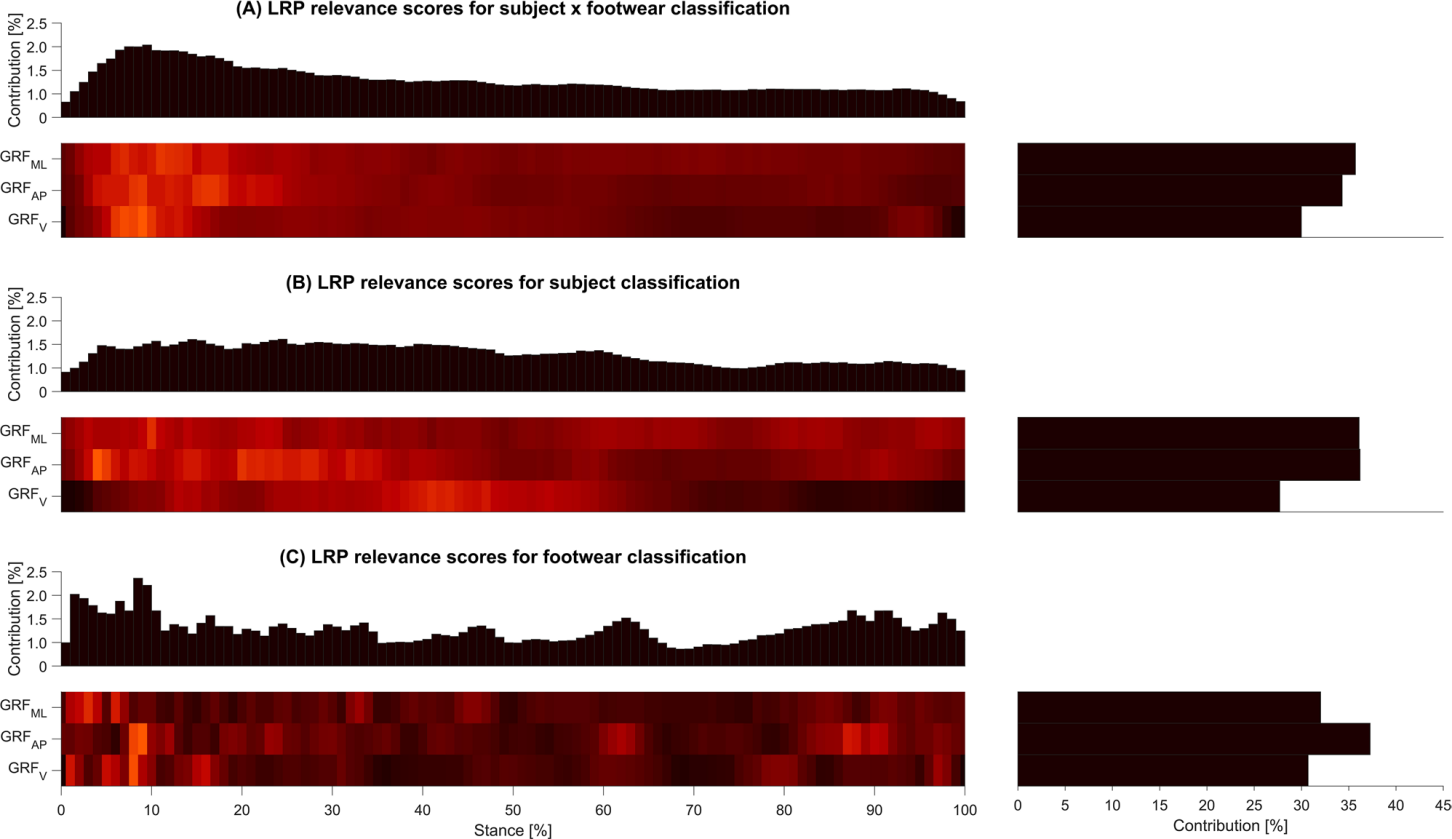
Input relevance evaluation of the machine learning models (model explanations) trained for the. employed classification tasks: (**A**) subject x footwear, (**B**) subject, (**C**) footwear. The input relevance scores were obtained by Layer-wise Relevance Propagation (LRP). For each subfigure (**A**–**C**): The top part on the left shows the summed contribution of the relevance scores for each of the 101 time points of the stance phase. In the bottom part on the left, lighter colours indicate variables of high relevance, while darker colours indicate variables of low relevance. The bottom right part highlights the summed contribution of relevance scores of each of the ground reaction forces (GRFs), namely mediolateral (GRF_ML_), anteroposterior (GRF_AP_), and vertical (GRF_V_). The figure was created using the MATLAB code provided by Hoitz et al. (2021)^18^.

Across all classification tasks, aggregated relevance scores of GRF_V_ were lowest (Fig. 4). Aggregated relevance scores of GRF_ML_ and GRF_AP_ were comparable in all but one classification tasks: within the footwear classification task, aggregated relevance scores of GRF_AP_ were substantially higher than aggregated scores of GRF_ML_ (Fig. 4C)*.*

The predictions of the SVM models in the subject x footwear classification task were predominantly based on the first 5–20% of the stance phase (Fig. 4A). For the subject classification task (Fig. 4B), regions with high summed relevance scores were more distributed across the stance phase. Regions of high relevance were observed at 0–40% (GRF_AP_) and at 25–60% (GRF_V_) of stance. In the footwear classification task, input values with high relevance scores were distributed across the entire stance phase with peak scores in the range of 0–10% and 80–100% (Fig. 4C).

**Figure 5:**
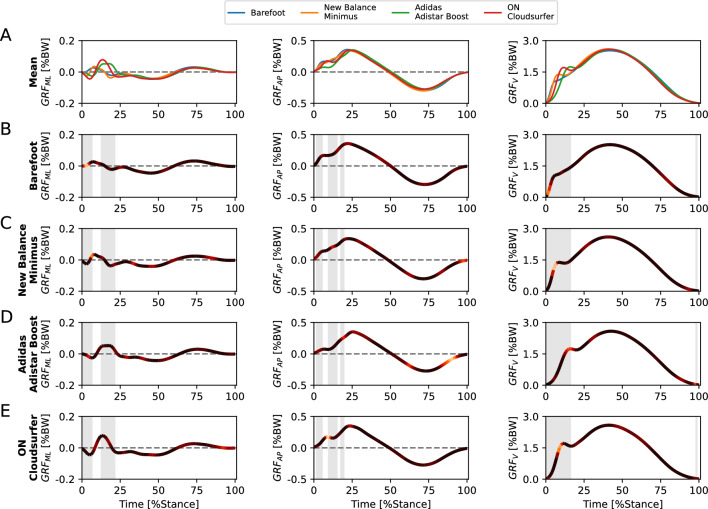
Input relevance evaluation of the machine learning models (class explanations) trained for the footwear classification task. (**A**) Mean ground reaction force (GRF) patterns including mediolateral (GRF_ML_), anteroposterior (GRF_AP_), and vertical (GRF_V_) of the four footwear conditions. (**B**–**E**) Mean GRFs of all test trials as a line plot for the class, color-coded via input relevance scores for the class obtained by Layer-wise Relevance Propagation (LRP). The grey-shaded areas highlight regions where statistical parametric mapping (SPM) indicated statistically significant differences between the four footwear conditions.

**Figure 6:**
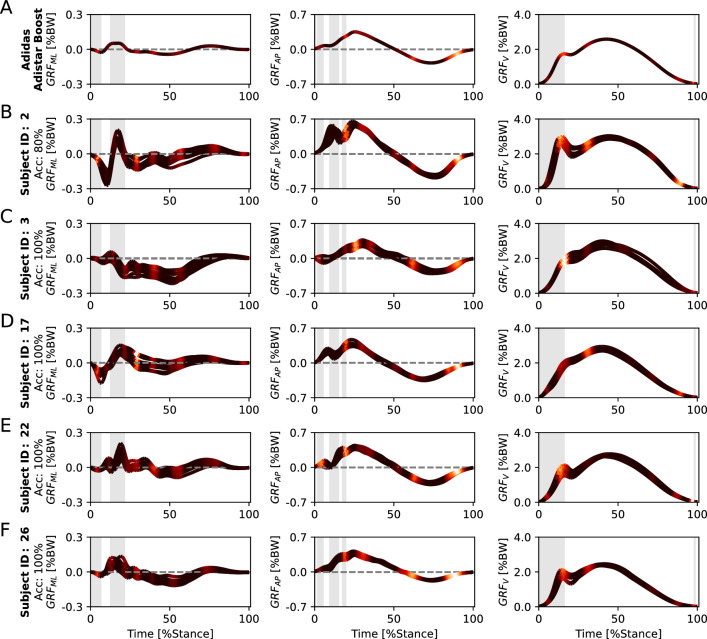
Input relevance evaluation of the machine learning models (subject explanations) trained for footwear classification task. (**A**) Mean ground reaction force (GRF) pattern including mediolateral (GRF_ML_), anteroposterior (GRF_AP_), and vertical (GRF_V_) of the footwear condition Adidas Adistar Boost, color-coded via input relevance scores obtained by Layer-wise Relevance Propagation (LRP). (**B**–**E**) Mean GRFs of all test trials as a line plot for one subject, color-coded via input relevance scores for the class obtained by Layer-wise Relevance Propagation (LRP). The grey-shaded areas highlight regions where statistical parametric mapping (SPM) indicated statistically significant differences between the four footwear conditions.

For the footwear classification task, we provide aggregated relevance scores for the four footwear conditions (class explanations) in Fig. 5 and examples of five subjects for the running shoe Adistar Boost (subject explanations) in Fig. 6. Subject examples for the remaining three footwear conditions can be found in the Supplementary Figs. 2, 3, and 4.

The Fig. 5 allows insights into GRF characteristics used by the SVM models for classifying different footwear conditions. The SVM models utilized class-specific characteristics in GRF_ML_, GRF_AP_, and GRF_V_ to distinguish the different footwear conditions. Notably, class-specific characteristics were prominent during the loading and impact phase (0–20%) of GRF_V_.

The Fig. 6 displays examples of GRF patterns with color-coded relevance scores of five subjects for the running shoe Adistar Boost in the footwear classification task. The figure highlights notable differences in the three GRF components between the five subjects, whereas the relevance scores focus on similar regions.

## Discussion

Movement patterns of thirty subjects that ran overground in three shod and one barefoot condition were analysed by a machine learning-based approach. Specifically, three-dimensional GRFs were classified using SVMs across three tasks: subject x footwear, subject, and footwear. The results for the different classification tasks will be discussed separately as they provide unique and novel insights into the field of running biomechanics.

### Subject x footwear classification

When movement patterns were classified according to the combination of subject and footwear condition, the accuracy of the SVM models was high (median accuracy: 96.2%). Hypothesis I, therefore, was supported by the findings of this work. This outcome suggests that each combination of runner and footwear results in a unique movement response that is identified by machine learning-based analysis. In other words, for each possible combination of runner and footwear, there is a high probability that a unique and identifiable movement pattern exists. This finding is supported by previous studies that reported inter-subject variability in responses to footwear^7,14^. Compared to previous studies, our results indicate that responses to footwear not only vary between subjects (e.g., some subjects show reduced GRF_V_ impact peaks in a shod compared to a barefoot condition, while other subjects show the opposite or no noticeable difference), but subjects also show distinct movement patterns. Future research needs to explore the (long-term) persistence of unique movement patterns for each combination of runner and footwear across various conditions, including multiple training sessions, extended running durations, different levels of fatigue, and varying running speeds.

The results presented in this study further reinforce the importance of considering subject-specific responses when examining the effects of footwear on biomechanical variables. This aligns with the earlier proposition made by Bates et al. (1983)^14^, which emphasized the significance of considering subject-specific responses in investigating the effects of footwear. These collective findings strongly advocate for a more comprehensive approach that acknowledges and incorporates individual variations in the study of footwear's impact on biomechanics.

### Subject classification (across footwear conditions)

When movement patterns were classified according to their respective subject, the SVM models achieved a median accuracy of 80%, supporting the second hypothesis of this work. This outcome implies that subjects expressed distinct movement characteristics, *regardless* of the tested footwear condition. An interpretation that is corroborated by previous findings that highlighted the presence of unique movement patterns for each tested individual in barefoot^15–17,25^ or personal (non-standardised) shod condition^18,19^. These distinct differences in movement patterns are likely related to individual anatomical characteristics^11,12^, muscle activation strategies^26,38^, and prevalence of previous injuries^13^.

Since subjects were recognised *regardless* of the footwear condition, one may speculate that subject-specific movement characteristics changed only minimally across the different running conditions. However, the degree to which a movement pattern can be maintained is runner specific (Fig. 3A). Some subjects demonstrated consistent movement pattern across different footwear conditions, while others showed high variations in the movement patterns across different footwear conditions. This is evident from the high classification accuracies of these runners (e.g., subjects 5, 12, 13, 25) compared to runners with low classification accuracies (e.g., subjects 10, 11). This is a finding that suggests different levels of sensitivity to footwear among subjects. Whether this sensitivity is restricted to footwear conditions, to distal body areas, or a general characteristic related to each individual needs further research. A runner's ability to maintain a movement signature under different running conditions may have implications for the association between footwear and performance (i.e., reduced energy consumption) as well as footwear and injury risk. For example, the impact of footwear on performance and injury risk may be greater in runners with different running patterns than in runners who maintain their running signature across different footwear conditions.

A reliable characterization of an individual's movement signature (i.e., encompassing the unique movement characteristics) requires accounting for a wide range of running conditions (e.g., different footwear types). This might not only account for the variations in movement patterns induced by distinctly different running conditions but also enables machine learning models to achieve better performance in subject classification, by effectively distinguishing and classifying individuals based on their specific movement characteristics and responses to different running conditions.

### Footwear classification (across subjects)

When movement patterns were classified according to their respective footwear condition, the SVM achieved a median accuracy of 87.8%. This outcome suggests that certain footwear-induced changes in movement patterns are similar *across* subjects. The highest prediction accuracy in the footwear classification task was found for the barefoot condition (Fig. 3B), suggesting that barefoot running is fundamentally different from shod running for most individuals; a finding supported by previous studies^20^. In addition, the confusion matrix in Supplementary Fig. 5 revealed that certain footwear conditions are more likely to be misclassified by the SVM, indicating stronger similarities between these footwear conditions. In the confusion matrix, we see that there is frequent confusion between barefoot running and running with the New Balance Minimus shoe (40 + 17 = 57 misclassifications), while Adidas Adistar Boost and ON Cloudsurfer are frequently confused (31 + 26 = 57 misclassifications).

The models' accuracy in the footwear classification task varied greatly across subjects (Fig. 3A). When individuals that were part of the test data have similar movement patterns in a footwear condition compared to individuals that were part of the training data, the SVM model could predict the correct footwear condition with a high level of accuracy. In comparison, when the movement patterns of individuals during testing were not similar to the movement patterns used during training, the classification performance dropped drastically. Hypothesis III, therefore, was partly supported by the findings of this work. Consequently, for some subjects, responses to a given footwear condition are comparable. These subjects may have similar functional needs towards footwear designs^2,23,24^. Given the finding of unique subject x footwear responses (subject x footwear classification in section "Subject x footwear classification"), future research needs to investigate to which extent subjects exhibit similar responses across different footwear conditions.

### Implications for footwear research

In this section, we compare the outcomes of the machine learning-based footwear classification with the results obtained from the group-based inference statistical evaluation, which is the commonly employed approach in biomechanical footwear research to assess differences between different footwear conditions. We discuss the implications, advantages, and challenges of using machine learning models for footwear classification and their potential contributions to advancing the field of biomechanical footwear research. In particular, we focus on the ability of machine learning models to consider individual differences in footwear research.

The SVM models utilized characteristics in GRF_ML_, GRF_AP_, and GRF_V_ to distinguish the different footwear conditions in the footwear classification task (Fig. 4C). GRF variables for which the statistical evaluation revealed a significant effect in the comparison of the four footwear conditions (Fig. 5 and Supplementary Table 1) were also utilized by the SVM models for the footwear classification task, as indicated by high LRP relevance scores (Fig. 5). In particular, the GRF_V_ during the loading and impact phase (0–20%) provided class-specific GRF characteristics that are relevant for the classification of the footwear conditions (Fig. 5). However, there is one exception regarding the temporal occurrence of the anterior peak of GRF_AP_, which did not exhibit increased relevance scores for the SVM models. This discrepancy suggests that the anterior peak of GRF_AP_ was statistically significant in differentiating the footwear conditions (Supplementary Table 1) but did not play a prominent role in the footwear classification performed by the SVM models. This may be due to the access of the SVM models to additional data before and after the peaks in the time-continuous variables or the fact that other GRF characteristics were identified to provide sufficient information for an accurate classification.

The SVM models based their predictions on GRF characteristics that exhibited statistically significant differences between footwear conditions, but also GRF characteristics that did not exhibit statistically significant differences (e.g., GRF_AP_ around 60% and 80–100% and GRF_ML_ around 45%, 60%, and 80–100% of the stance phase). This indicates that machine learning models learned additional GRF characteristics relevant for the footwear classification task. Possible implications of this observation will be further discussed in the following two subsections.

#### Machine learning models for footwear classification across subjects

The subject explanations in Fig. 6 illustrate that SVM models could predict the correct footwear condition (i.e., Adidas Adistar Boost) for a variety of different movement patterns (from different subjects), which differed from the mean curve of the footwear condition. Machine learning models have the capability to consider various responses to footwear from the training data when making predictions. This indicates that machine learning models can learn different representations (movement patterns) for a specific footwear condition allowing for a more versatile and nuanced mapping of the footwear effect on running patterns. Thus, machine learning models may have the potential to consider variances in movement patterns related to relevant subgroups in the dataset (e.g., forefoot, midfoot, and rearfoot runners, male and female runners, or novice, recreational, and high-calibre runners) without requiring explicit prior knowledge about them. Influencing factors that were previously less considered, such as functional groups, may also be implicitly modelled by the SVM models^39^.

However, this is only possible when one subject that was part of the training data has similar movement patterns in this footwear condition. The performance of machine learning models (highly) depends on the data used to train them. In the context of footwear classification this means that the machine learning models can predict the correct footwear condition if the data of a tested subject is similar to (and from the same distribution as) a subject that was part of the training data. However, if this is not the case, the SVM model is not able to accurately predict the correct footwear condition. Our experiments showcase that the data from a crucial minimum number of subjects seems to be required to train machine learning models that can represent a wide range of individuals (Supplementary Fig. 6). When the number of subjects is relatively small, as often is the case in biomechanical studies to date, there is a higher risk that the running patterns of individual subjects cannot be modelled well (e.g., subject 13 in our study). A representative database containing a large and diverse number of subjects could, however, not only allow to consider the above-mentioned subgroups, but even allows the analysis on an individual level using the most similar subject in the database in the sense of a “digital twin”. This could have a great potential especially for the prediction of long-term effects of footwear for individual subjects.

#### Subject-specific machine learning models for footwear classification

Two findings of our study, (I) the identification of unique movement patterns for each combination of runner and footwear that provided evidence for subject-specific responses to footwear (subject x footwear classification in section "Subject x footwear classification") and (II) subject-specific movement characteristics that changed only minimally across the different running conditions in most subjects (subject classification in section "Subject classification (across footwear conditions)") , support research strategies that assess the effects of footwear at the individual level, i.e., *single-subject* approaches^22^.

Machine learning models for footwear classification can also be trained on data from one individual subject (subject-specific model). Subject-specific machine learning models could be used to predict which footwear design exhibits movement patterns that are most similar to a desired reference movement pattern (e.g., barefoot running, running with the personal shoe, preferred movement path^3,20,40^, and habitual movement path^41,42^) for an individual subject. In this context, machine learning models have the advantage that they can provide subject-specific predictions based on various multi-dimensional and time-continuous (biomechanical) variables without requiring explicit threshold definitions. For example, subject-specific machine learning models can be used in randomised controlled trials or running stores to predict the footwear that best matches the runner’s movement patterns.

### Future research

The present study employed GRF data during running to demonstrate how machine learning models (i.e., SVMs) combined with explainable artificial intelligence methods (i.e., LRP) can enrich the evaluation of footwear effects.

Future research needs to corroborate the presented findings by incorporating more diverse and larger datasets and experimental settings by considering e.g., on following four aspects. Firstly, a larger and more diverse population should be included, encompassing anthropometric and demographic factors like sex, age, and running skill level. Secondly, further investigations including preferred, fixed, and systematically altered running speeds conditions are necessary to examine dependencies between the obtained classification results and running speed in more detail. Thirdly, it is crucial to incorporate data collected during “real-world” scenarios, such as long-distance runs in common training or competition settings with variable influences (e.g., according to circadian rhythms, menstrual cycle, fatigue, mood, and running surface). Assessing the effect of footwear in these authentic contexts provides insights beyond controlled laboratory conditions, offering a more realistic evaluation. Lastly, future research should also incorporate specifically selected footwear conditions, including variations like different shoe types, cushioning levels, and support features.

With a larger dataset, the utilization of more complex machine learning models, such as deep learning architectures, holds the potential to yield deeper insights and improve the classification performance. Additionally, to ensure robustness and generalizability of the machine learning models, it is advisable to utilize a hold-out approach where a completely unseen test dataset is used for evaluation. Due to the limited size of the employed dataset, the use of k-fold cross-validation proved to be appropriate to obtain robust results. However, a larger dataset would allow for a more reliable assessment of the model's performance on unseen test data.

While GRFs seem to be particularly suitable for generating such larger-scale datasets described above (i.e., as they are considered to be reliable, also across different laboratories) and have performed very well in the present experiments, it is important to acknowledge that GRFs are integral variables that aggregate the accelerations of the centre of mass of all body segments. Given that predictions of machine learning models for footwear classification are characterised by a plethora of versatile features (Fig. 4C), our findings imply that considering multi-dimensional and time-continuous biomechanical data such as full-body kinematics and kinetics appear to be promising for evaluations of (subject-specific) footwear effects. Future research involving a combination of bilateral kinematic, kinetic, and electromyographic data is needed to relate the presented results to a functional perspective.

## Conclusion

The present findings suggest that unique movement signatures (across footwear) and unique responses to each footwear design can be modelled for each subject. Our results support the hypothesis that considering subject-specific responses is advantageous for a better understanding of the functional effects of footwear during running. The incorporation of different multi-dimensional and time-continuous biomechanical data (e.g., whole body kinematics and kinetics) seems to be similarly auspicious for a more differentiated (subject-specific) evaluation of the effects of footwear. Machine learning methods seem to be a promising and valuable extension to previous (subject-specific) approaches for footwear evaluation and recommendation.

## Supplementary Information

Supplementary Information.

## Data Availability

The dataset generated during and/or analysed during the current study are available from the corresponding author on reasonable request.
