# Supplementary material for: Identification of subject-specific responses to footwear during running
Source: Sci Rep. 2023 Jul 12;13:11284. doi: 10.1038/s41598-023-38090-0 (PMC10338529; doi:10.1038/s41598-023-38090-0)
Supplement: Supplementary file 1 — Supplementary Information. [file 41598_2023_38090_MOESM1_ESM.pdf]

Identification of Subject-specific Responses to Footwear During Running

-

SUPPLEMENTARY MATERIAL

Fabian Horst[1], Fabian Hoitz[2,3], Djordje Slijepcevic[4], Nicolas Schons[1], Hendrik Beckmann[1], Benno M. Nigg[3] and Wolfgang I. Schöllhorn[1]

[1]Department of Training and Movement Science, Institute of Sport Science, Johannes Gutenberg-University Mainz, Mainz, Germany

[2]Biomedical Engineering, Schulich School of Engineering, University of Calgary, Calgary, Alberta, Canada

[3]Human Performance Laboratory, Faculty of Kinesiology, University of Calgary, Calgary, Alberta, Canada

[4]Institute of Creative Media Technologies, Department of Media & Digital Technologies, St. Pölten University of Applied Sciences, St. Pölten, Austria

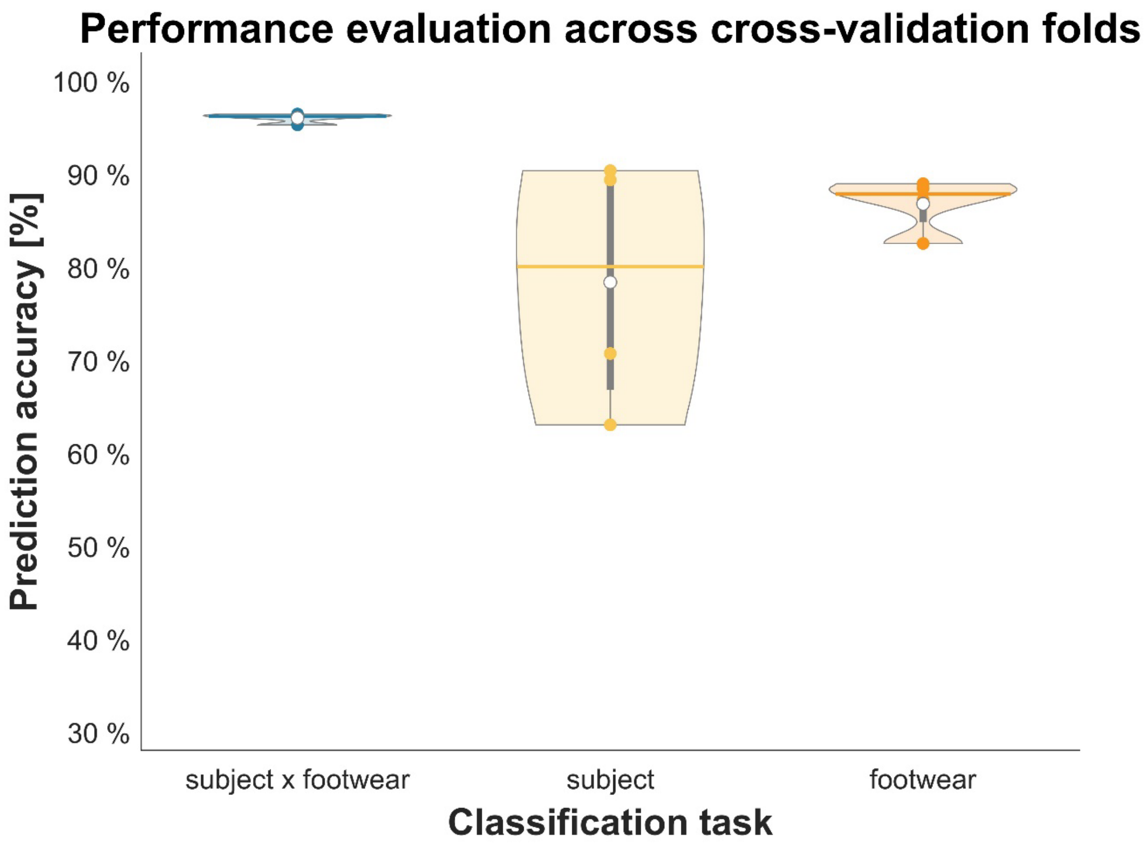

Supplementary Figure 1. *Fold-wise evaluation of the support vector machine model performance. Performance evaluation of the models trained for the three employed classification tasks: subject x footwear (in blue on the left), subject (in yellow in the middle), and footwear (in orange on the right). The task-specific zero-rule baseline values are 0.8% (subject x footwear classification), 3.3% (subject classification), and 25.0% (footwear classification). The prediction accuracy is shown as violin plots with median (solid line), mean (white dot), interquartile range from Q1 to Q3 (grey bar), and individual values (coloured dots) of the prediction accuracy for individual folds. The figure was created using the MATLAB code provided by Bechtold et al. (2022).*

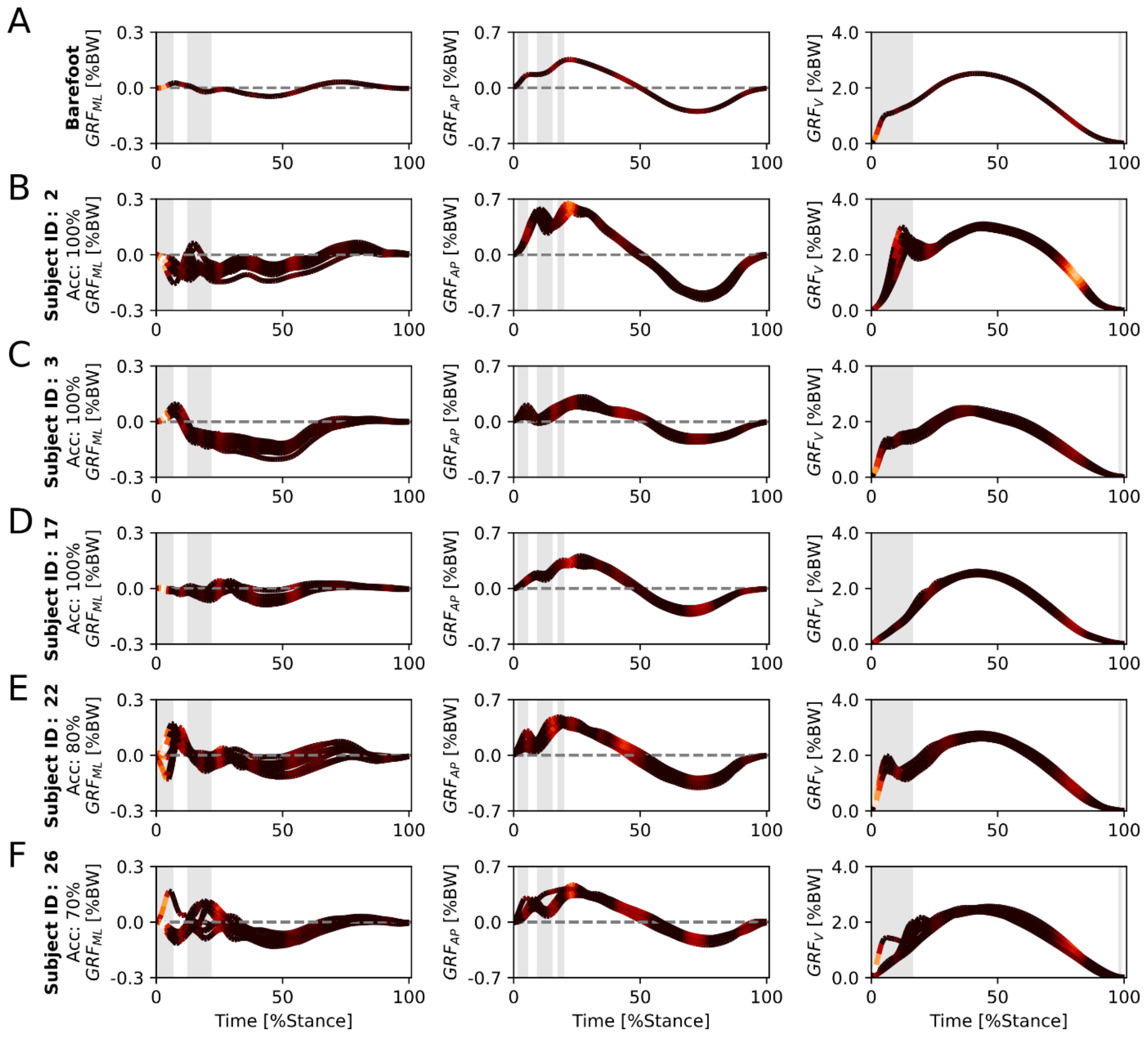

Supplementary Figure 2. *Input relevance evaluation of the machine learning models (subject explanations) trained for footwear classification task.* ***(A)*** *Mean ground reaction force (GRF) pattern including mediolateral (GRF_{ML}), anteroposterior (GRF_{AP}), and vertical (GRF_{V}) of the footwear condition Barefoot, color-coded via input relevance scores for the class obtained by Layer-wise Relevance Propagation (LRP).* ***(B-E)*** *Mean GRFs of all test trials as a line plot for one subject, color-coded via input relevance scores for the class obtained by Layer-wise Relevance Propagation (LRP). The grey-shaded areas highlight regions where statistical parametric mapping (SPM) indicated statistically significant differences between the four footwear conditions.*

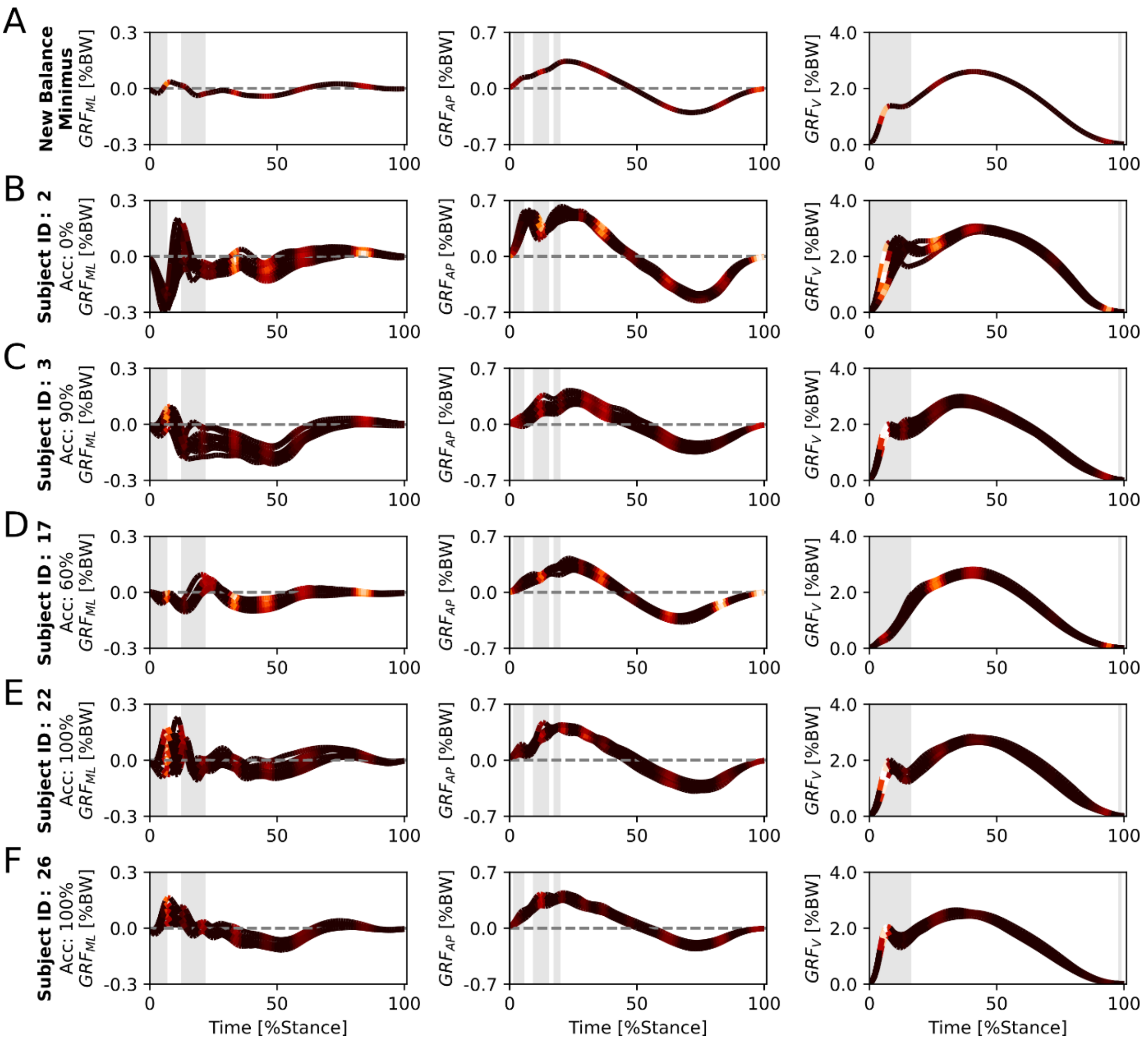

Supplementary Figure 3. *Input relevance evaluation of the machine learning models (subject explanations) trained for footwear classification task.* ***(A)*** *Mean ground reaction force (GRF) pattern including mediolateral (GRF_{ML}), anteroposterior (GRF_{AP}), and vertical (GRF_{V}) of the footwear condition New Balance Minimus, color-coded via input relevance scores for the class obtained by Layer-wise Relevance Propagation (LRP).* ***(B-E)*** *Mean GRFs of all test trials as a line plot for one subject, color-coded via input relevance scores for the class obtained by Layer-wise Relevance Propagation (LRP). The grey-shaded areas highlight regions where statistical parametric mapping (SPM) indicated statistically significant differences between the four footwear conditions.*

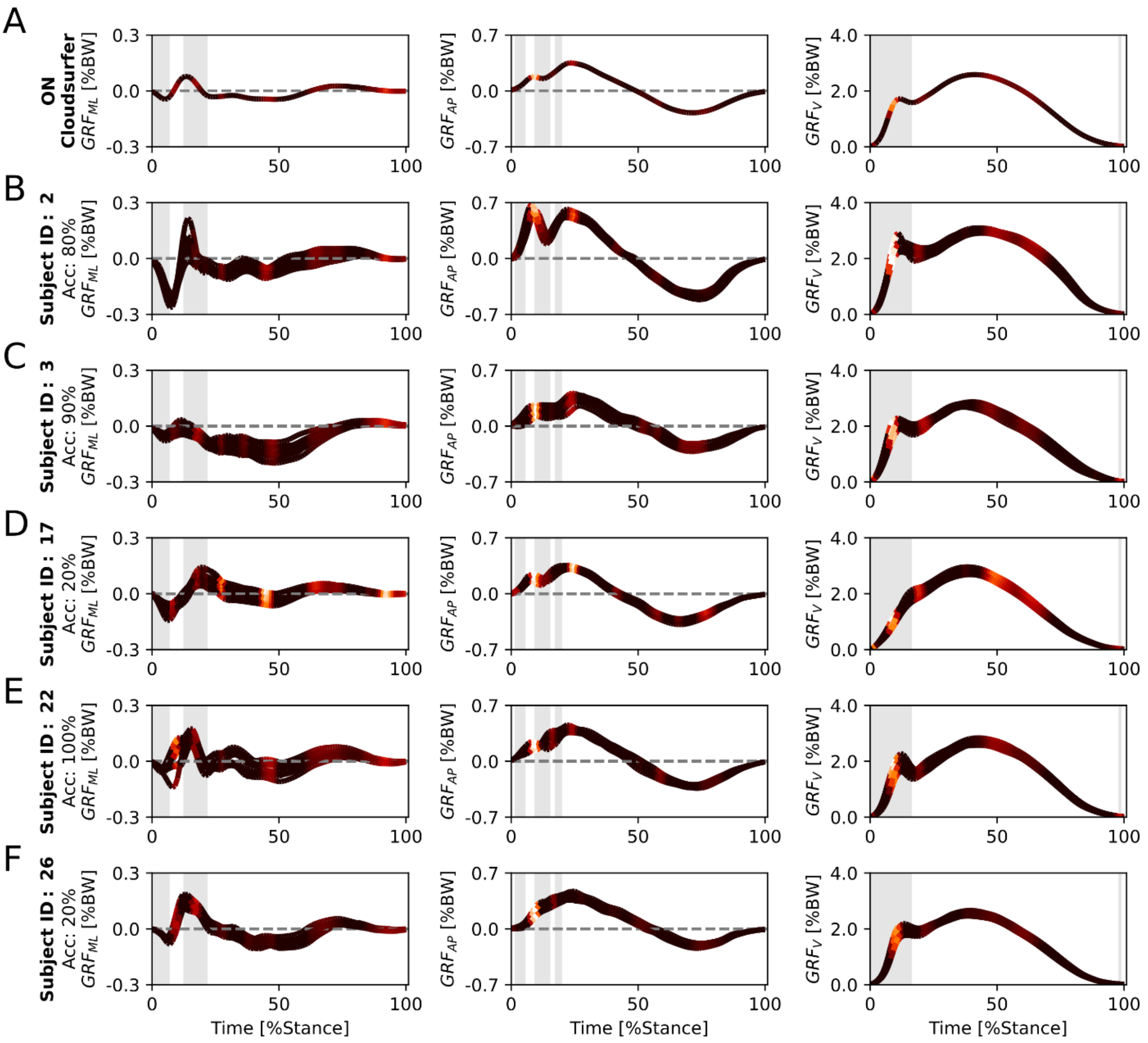

Supplementary Figure 4. *Input relevance evaluation of the machine learning models (subject explanations) trained for footwear classification task.* ***(A)*** *Mean ground reaction force (GRF) pattern including mediolateral (GRF_{ML}), anteroposterior (GRF_{AP}), and vertical (GRF_{V}) of the footwear condition ON Cloudsurfer, color-coded via input relevance scores for the class obtained by Layer-wise Relevance Propagation (LRP).* ***(B-E)*** *Mean GRFs of all test trials as a line plot for one subject, color-coded via input relevance scores for the class obtained by Layer-wise Relevance Propagation (LRP). The grey-shaded areas highlight regions where statistical parametric mapping (SPM) indicated statistically significant differences between the four footwear conditions.*

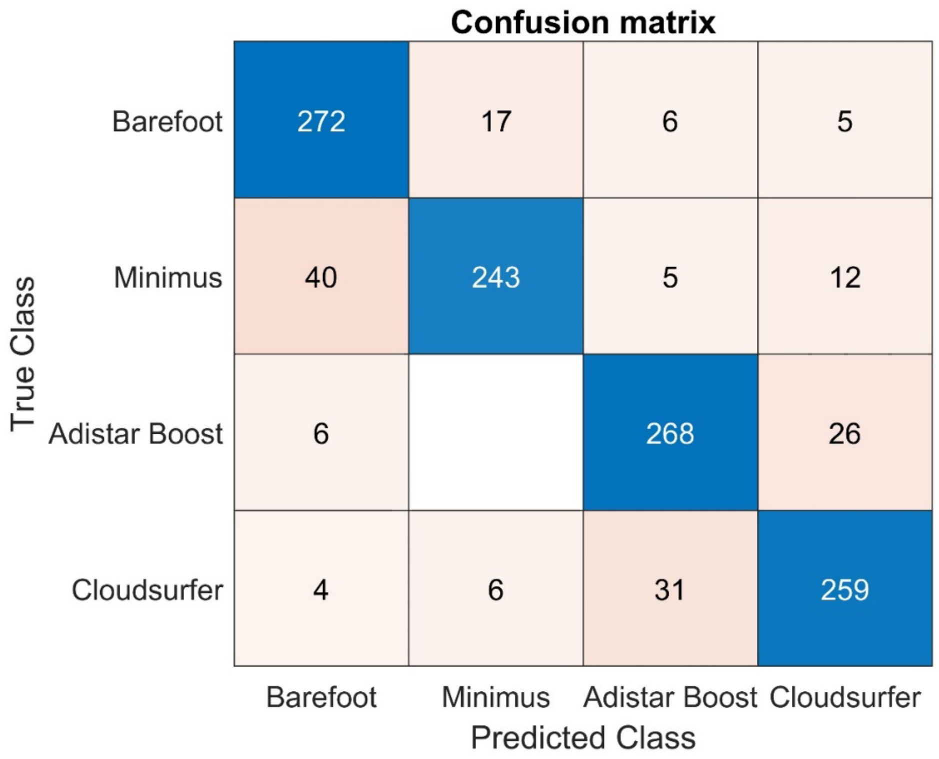

Supplementary Figure 5. *Confusion matrix of support vector machine (SVM) models trained for the footwear classification task.*

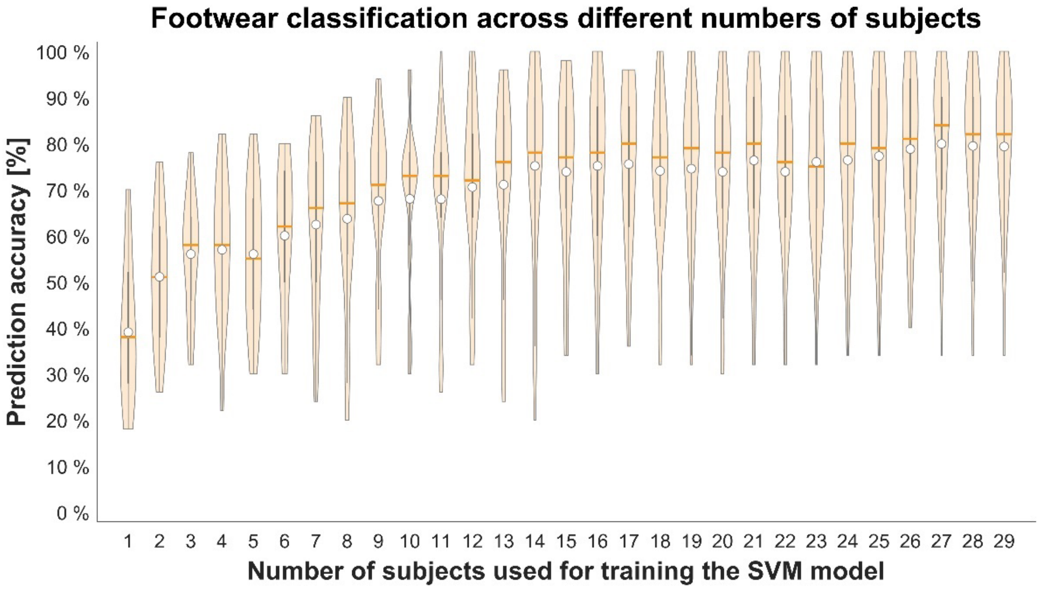

Supplementary Figure 6. *Performance evaluation of an increasing number of subjects used for training of support vector machine (SVM) models for the footwear classification task. This method involved progressively increasing the number of subjects used for training. By employing this iterative strategy, we could investigate how varying subject quantities influenced the classification performance. The prediction accuracy was obtained using a leave-subject-out cross-validation configuration, presented as violin plots displaying the median (solid line), mean (white dot), and interquartile range from Q1 to Q3 (grey bar). The figure was created using the MATLAB code provided by Bechtold et al. (2022).*

Supplementary Table 1. *Statistical evaluation of differences in time-discrete ground reaction force (GRF) variables between the four footwear conditions (Barefoot, New Balance Minimus, Adidas Adistar Boost, and ON Cloudsurfer).*

GRF variable	Barefoot (1)	New Balance Minimus (2)	Adidas Adistar Boost (3)	ON Cloudsurfer (4)	χ^2	df	p	ε^2	Post-hoc
**Contact time [s]**	0.253 (0.239-0.279)	0.258 (0.239-0.287)	0.265 (0.252-0.294)	0.267 (0.248-0.295)	3.80	3	0.283	0.03	-
**GRF_{ML} medial peak [%BW]**	0.09 (0.06-0.12)	0.09 (0.06-0.14)	0.09 (0.05-0.14)	0.11 (0.06-0.14)	0.87	3	0.833	0.01	-
**GRF_{ML} medial peak [%stance]**	0.12 (0.08-0.28)	0.11 (0.08-0.25)	0.15 (0.14-0.19)	0.14 (0.13-0.16)	7.27	3	0.064	0.06	-
**GRF_{ML} lateral peak [%BW]**	0.08 (0.06-0.10)	0.08 (0.07-0.10)	0.08 (0.06-0.10)	0.09 (0.07-0.10)	1.57	3	0.667	0.01	-
**GRF_{ML} lateral peak [%stance]**	0.24 (0.11-0.48)	0.28 (0.18-0.41)	0.44 (0.25-0.51)	0.28 (0.10-0.44)	5.55	3	0.136	0.05	-
**GRF_{AP} posterior peak [%BW]**	0.37 (0.34-0.44)	0.35 (0.31-0.39)	0.36 (0.32-0.42)	0.35 (0.31-0.42)	2.74	3	0.434	0.02	-
**GRF_{AP} posterior peak [%stance]**	0.22 (0.20-0.23)	0.23 (0.21-0.25)	0.25 (0.24-0.27)	0.23 (0.22-0.25)	27.13	3	< .001	0.23	1-3 W=6.69 p<0.001<br>1-4 W=3.64 p=0.050<br>2-3 W=4.66 p=0.005<br>3-4 W=4.62 p=0.006
**GRF_{AP} anterior peak [%BW]**	0.26 (0.24-0.36)	0.28 (0.24-0.38)	0.26 (0.22-0.33)	0.26 (0.23-0.33)	3.13	3	0.372	0.03	-
**GRF_{AP} anterior peak [%stance]**	0.73 (0.72-0.74)	0.72 (0.70-0.73)	0.74 (0.72-0.75)	0.71 (0.70-0.73)	13.03	3	0.005	0.11	2-3 W=3.85 p=0.033<br>3-4 W=4.47 p=0.008
**GRF_V active peak [%BW]**	2.48 (2.30-2.74)	2.57 (2.43-2.82)	2.59 (2.39-2.81)	2.57 (2.41-2.79)	1.72	3	0.634	0.01	-
**GRF_V active peak [%stance]**	0.42 (0.40-0.44)	0.41 (0.39-0.43)	0.42 (0.41-0.45)	0.41 (0.40-0.43)	6.49	3	0.090	0.06	-
**GRF_V impact peak [%BW]**	1.49 (1.21-1.73)	1.56 (1.24-1.77)	1.76 (1.46-1.86)	1.81 (1.48-1.99)	12.47	3	0.006	0.11	1-4 W=3.81 p=0.036<br>2-4 W=3.70 p=0.044
**GRF_V impact peak [%stance]**	0.07 (0.06-0.13)	0.09 (0.07-0.11)	0.16 (0.14-0.17)	0.12 (0.11-0.13)	56.87	3	< .001	0.48	1-3 W=7.61 p<0.001<br>2-3 W=8.66 p<0.001<br>2-4 W=5.12 p=0.002<br>3-4 W=8.36 p<0.001
**GRF_V avg. loading rate [%BW/s]**	104.4 (50.9-117.4)	97.1 (69.2-114.7)	51.7 (45.6-61.5)	76.1 (59.6-93.6)	21.87	3	< .001	0.18	1-3 W=4.52 p=0.008<br>2-3 W=5.39 p<0.001<br>3-4 W=5.69 p<0.001
**GRF_V inst. loading rate [%BW/s]**	134.1 (99.9-162.0)	128.5 (91.7-149.3)	83.3 (61.8-95.2)	112.1 (87.9-135.9)	20.22	3	< .001	0.17	1-3 W=5.33 p<0.001<br>2-3 W=4.70 p=0.005<br>3-4 W=4.83 p=0.004
